# The impact of our fight

**DOI:** 10.1002/cnr2.1563

**Published:** 2021-10-06

**Authors:** Sebastián Andrés Palacios M., María Gabriela Palacios M., Rafael Francisco Palacios B.

**Affiliations:** ^1^ Contabilidad y Auditoría Universidad Politécnica Salesiana Guayaquil Ecuador; ^2^ Facultad de Medicina Universidad de Guayaquil Guayaquil Ecuador; ^3^ Periodismo Universidad Laica Vicente Rocafuerte Guayaquil Ecuador

**Keywords:** APNACC, cancer, fight, warriors

## Abstract

The association of parents of children and adolescents with cancer (APNACC), is a nongovernmental organization law institution, with legal status, nonprofit and social aid, whose mission, vision and objectives is to ensure the rights of the children and adolescents so that they are not violated, in addition to guiding and directing parents who enter this world of childhood and adolescent cancer, because we believe that addressing in time saves lives and optimizes resources. Through this article, it is intended to describe the impact that APNACC has had on this cause, the fight against childhood and adolescent cancer; from achieving a state law for free care for catastrophic and orphan or rare diseases, to obtaining approval of a basic list of medications.

## 1 INTRODUCTION

The association of parents of children and adolescents with cancer, otherwise known as the Asociación de Padres de Niños y Adolescentes con Cáncer (APNACC), is a nongovernmental organization (NGO) institution of law, with legal status for the provision of nonprofit and social aid in Guayaquil, Ecuador. APNACC was established May 30, 2018 (Resolution N° MIES‐CZ‐8‐DDG2‐2018‐0051‐R) for the purpose of ensuring the rights of children and adolescents with cancer are not violated by state law. We maintain the state accountable for covering the costs of treating cancer from the onset of diagnosis and throughout all treatment stages through advocacy and lobbying. We support the children and adolescents with cancer and their families in their fight against cancer. We refer to our brave children as “warriors” as they are fighting a disease that attacks their body and must fight to save their life. In addition to guiding and directing the parents of these children and adolescents, we offer psychosocial‐oncological assistance projects and strategic alliances with key stakeholders from the public and private sectors.

The objective of this article is to describe the contribution and impact of APNACC.

## 2 THE REALITY OF THE PROBLEM

In our country, there are no defined public policies regarding cancer, due to this, the Government does not take full responsibility to exercise them in practice. These policies should be elaborated by true specialists and experts such as pediatric oncologists, legal specialists in public policy, oncology hospitals and accredited nongovernmental organizations with experience in the subject.

However, there are recognized organizations in the country that have no vocation of service, do not contribute positively to our cause, downplaying activism in the fight against childhood cancer.

Likewise, public officials become an obstacle by having limited power and little or no knowledge about childhood cancer, to influence any decision or change.

## 3 THE BEGINNING OF A FIGHT

APNACC arises from a group of committed parents of children with cancer, initially united in a committee, which was convened in 2011, and mobilized their networks and resources to advocate for change, dignified and fair care for their children.

After much effort, a dialogue was started with the representatives of the government. We managed to sensitize the president of the nation at that time, to incorporate CHAPTER III‐A OF CATASTROPHIC AND RARE OR ORPHAN DISEASES, in the Law Organic Health through Law No. 0 that was published in Official Gazette 625 of January 24, 2012.^1^

As result, effective from January 2012, all catastrophic, rare, or orphan diseases are treated, free of charge. This right is granted through the comprehensive Public Health Network (RPIS)–Ministry of Public Health (MSP), and the private server, the Society for the Fight Against Cancer (SOLCA), in all its treatment protocols, in hospitalization or on an outpatient basis.

It should be noted that there are patients treated by the RPIS who are referred to SOLCA, as well as those who are treated through government insurance Ecuadorian Social Security Institute (IESS), Social Security Institute of the National Police (ISSPOL), and Institute of Social Security of the Armed Forces of Ecuador (ISSFA). The government insurances are IESS, ISSPOL, and ISSFA.

Likewise, the RPIS has two pediatric specialty hospitals with oncology areas implemented, but not complemented; in the city of Quito, Baca Ortiz Hospital, and in Guayaquil, Dr. Francisco de Icaza Bustamante Hospital. The latter treats the majority of patients from the 24 provinces in the country.

## 4 ACTIVISM FOR THE CAUSE

As a result of our first achievement, the state law for the gratuity of catastrophic and rare or orphan diseases, we decided to carry out more activism in this medium.

**FIGURE 1:**
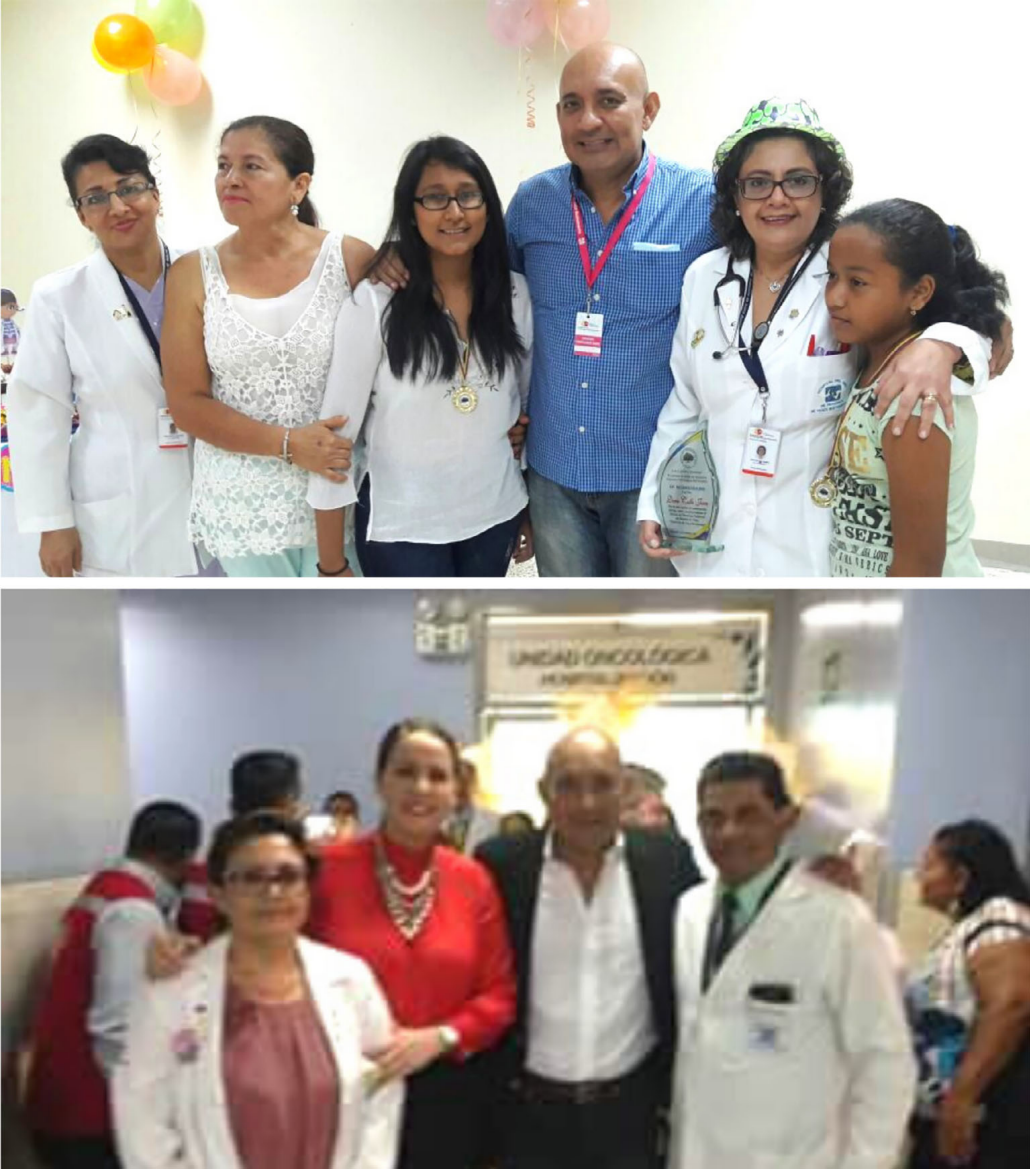
Opening (above, 2016) and repowering (below, 2017) of the oncology unit

In 2016, we contributed to the inauguration of the first Oncology Unit at Dr. Francisco De Icaza Bustamante pediatric hospital in Guayaquil (Figure 1) and in subsequent year led efforts to repower of its capacities. This initiative to repowering the oncology unit was promoted by the unit's leader of that time.

It is worth mentioning that initially it was an outpatient ward, and for the first time, there was on the part of the MSP, a unit implemented but not complemented, in the service of children and adolescents with cancer. That is to say, although it is possible to provide chemotherapy protocols, it does not have other equipment and services, for example, radiotherapy, positron emission tomography (PET) scan, among others.

When we realized that we were a support tool for parents and their warriors, we felt that “APNACC is love for the cause,” a phrase that we later adopted as the slogan of our organization.

**FIGURE 2:**
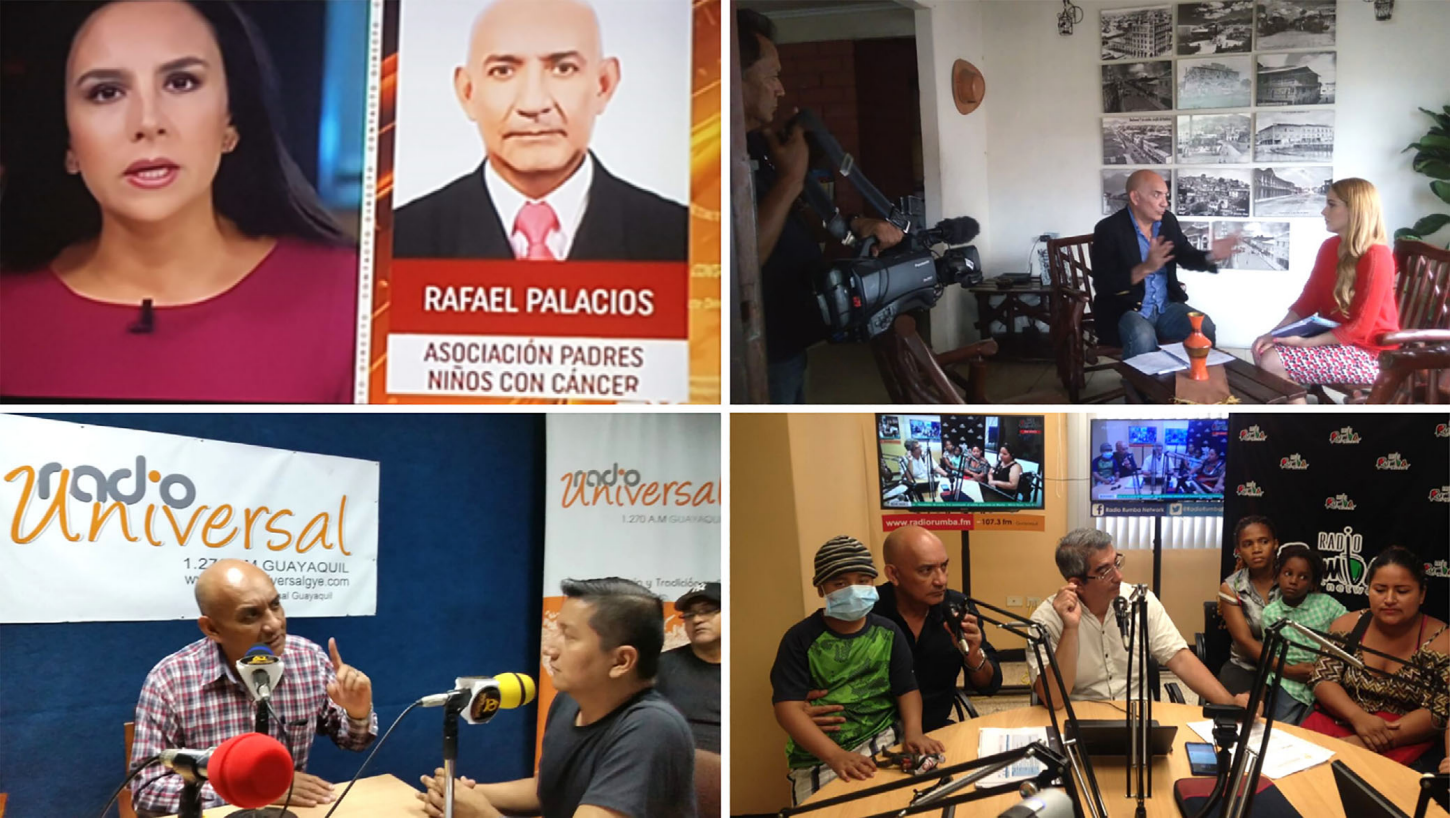
Approach in the national media

It was then when we decided to establish ourselves legally in May 2018, to make this cause visible and to get a greater commitment from the authorities of the country. To make ourselves more noticeable and effective, we actively engaged with several media channels, newspapers, radio, national television, and even social networks (Figure 2).

**FIGURE 3:**
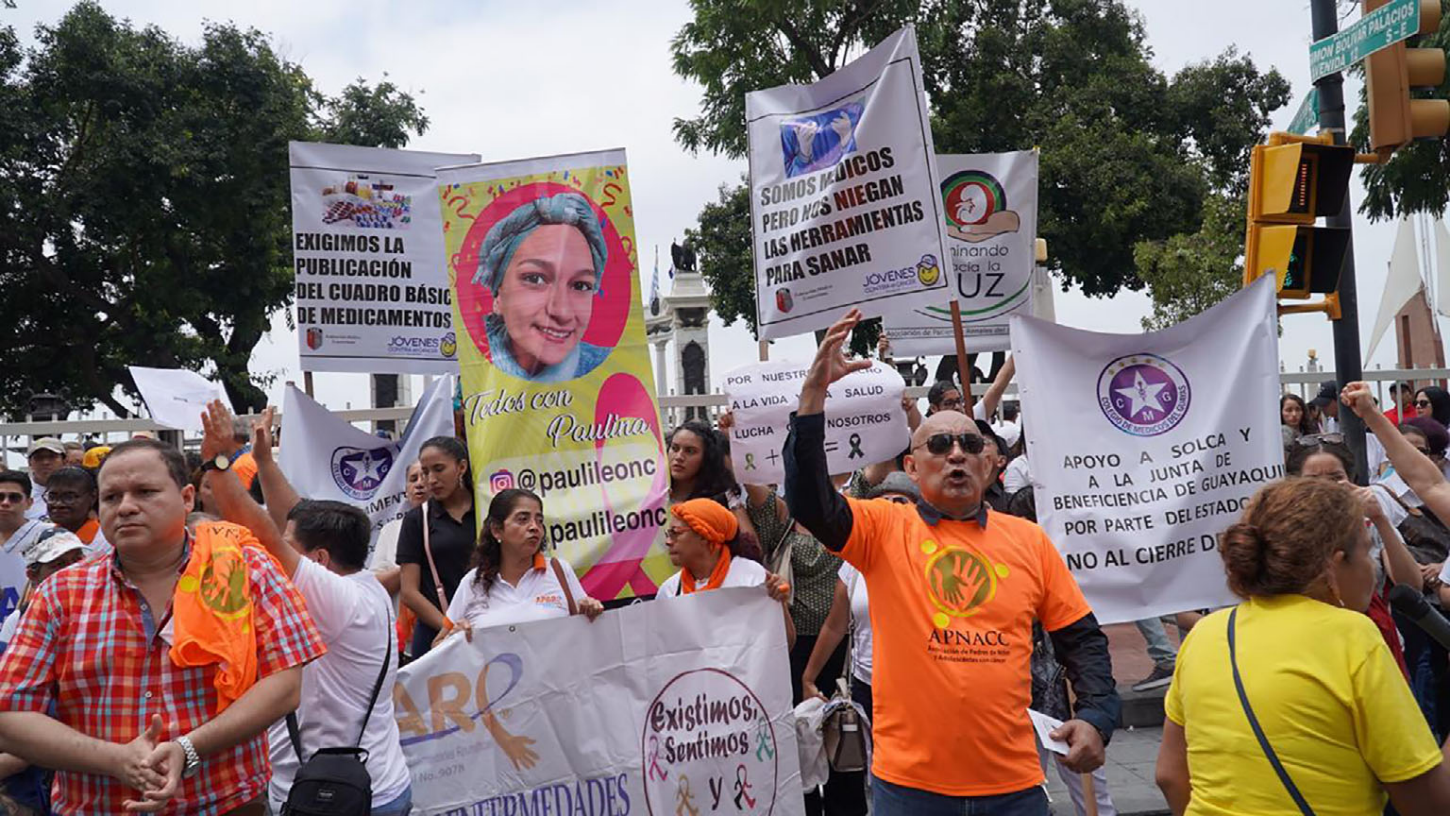
March to demand the basic table of medicines

The initial purpose to make a change in a small group of parents, expanded to include many more with children with other pathologies, who saw this as a new opportunity to stand up and demand from the State approval of Basic Table of Medicines for catastrophic and rare or orphan diseases (Figure 3).

**FIGURE 4:**
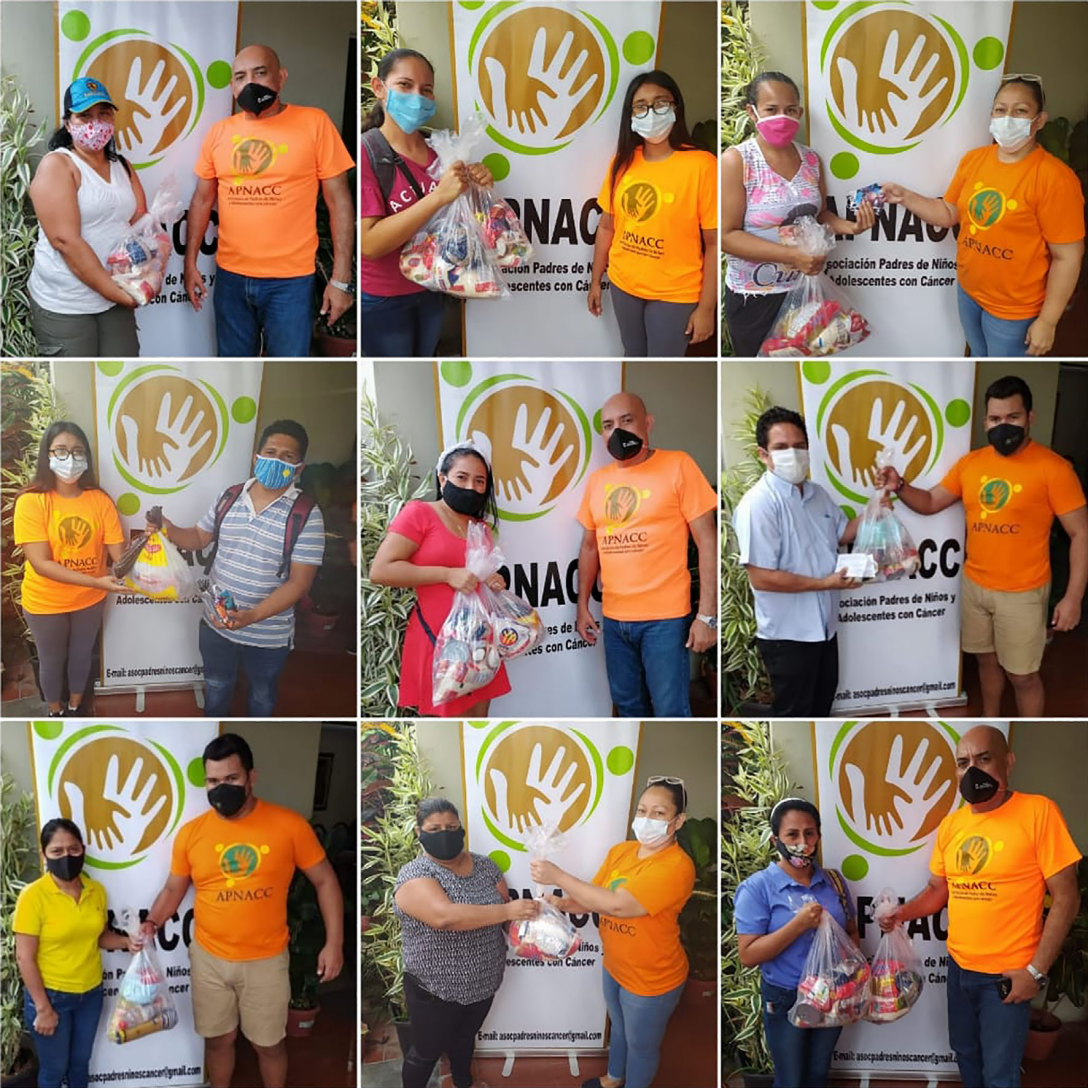
Distribution of food resources

During the COVID‐19 pandemic, realizing the limitations of virtual meetings, we saw an opportunity to forge a strategic alliance with the Diakonía Food Bank, who provided us with basic food resources for the warriors' families (Figure 4).

## 5 A REAL CHANGE

Throughout these years and in accordance with the events reported, we have carried out activism to continue ensuring that the rights of children and adolescents are not violated. However, we believe that the State should guarantee us optimal care and access to quality medications without the need for organizations and advocates like ourselves to go out on the street and protest because certain regulations are not being complied with.

As a solution to these irregularities, we are looking for:

- The law of childhood cancer Ecuador – Ley del Cáncer Infantil Ya Ecuador.^2^
- Strategic alliance with the MSP to be observers as an NGO knowledgeable on the topic.
- Social and Psycho‐oncological Assistance Center for warriors.

It is important to highlight that the lack of resources and support from the country's authorities and civil society has not allowed us to advance but nonetheless, we continue our fight to achieve these objectives.

## 6 CONCLUSION

Although much remains to be done, we believe that what has been achieved is of the utmost importance for this cause, because the word cancer is synonymous to death when the necessary resources are not available. However, having access to a law that allows free care and essential medicines for treatment protocols, means a possibility of life for our children.

Our activism has not only benefited childhood and adolescent cancer, but also adult cancer and other pathologies.

We are sure that timely addressing of patients' needs saves lives and optimizes resources, not only for patients but also for the State.

We will continue working and seek more alliances, to continue fighting for the rights of our children and adolescents with cancer.

## CONFLICT OF INTEREST

The authors have declared no conflict of interest.

## AUTHOR CONTRIBUTIONS

**Rafael Palacios Bravo:** Conceptualization (equal); writing – original draft (lead); supervision (supporting). **Sebastián Palacios Muñoz:** Conceptualization (equal); formal analysis (equal); investigation (lead); methodology (lead); project administration (equal); resources (supporting); software (lead); supervision (supporting); visualization (equal); writing – original draft (lead); writing – review and editing (lead). **María Palacios Muñoz:** Writing – review and editing (supporting); visualization (equal).

## ETHICS STATEMENT

All procedures followed were in accordance with the ethical standards of the Helsinki Declaration of 1975.

## Data Availability

Data sharing is not applicable to this article as no new data were created or analyzed in this study.
